# Reducing Inequities in Early Childhood Mental Health: How Might the Neighborhood Built Environment Help Close the Gap? A Systematic Search and Critical Review

**DOI:** 10.3390/ijerph16091516

**Published:** 2019-04-29

**Authors:** Amanda Alderton, Karen Villanueva, Meredith O’Connor, Claire Boulangé, Hannah Badland

**Affiliations:** 1 Centre for Urban Research, RMIT University, Melbourne 3000, Australia; karen.villanueva@rmit.edu.au (K.V.); claire.boulange@rmit.edu.au (C.B.); hannah.badland@rmit.edu.au (H.B.); 2 Centre for Community Child Health, Murdoch Children’s Research Institute, Royal Children’s Hospital, Melbourne 3052, Australia; meredith.oconnor@mcri.edu.au; 3 Department of Paediatrics, University of Melbourne, Melbourne 3052, Australia; 4 ANU Centre for Social Research and Methods, The Australian National University, Canberra 0200, Australia

**Keywords:** children, health inequalities, mental health, neighborhood effects, social determinants, socio-ecological model, urban planning

## Abstract

Background: Optimal mental health in early childhood is key to later mental health, physical health, education, and social outcomes; yet, children facing disadvantage tend to have worse mental health and fewer opportunities to develop this foundation. An emerging body of research shows that neighborhoods provide important opportunities for the development of children’s mental health. Synthesizing this evidence can advance understandings of the features of the neighborhood built environment (e.g., housing, parks) that (1) promote optimal mental health in childhood and (2) reduce mental health inequities. Methods: We systematically searched and critically reviewed the international quantitative literature investigating associations between the neighborhood built environment and young children’s mental health. Results: 14 articles met inclusion criteria; most examined nature or public open space. Studies tended to find greater access to or quantity of neighborhood nature or public open space were associated with better mental health. Significant gaps included a lack of studies investigating social infrastructure, and few studies examined how the built environment related to positive mental health (i.e., functioning, rather than problems). Conclusions: Current evidence suggests there is some relationship, but additional research is needed that addresses these gaps and examines differences in associations between child subgroups (e.g., diverse socioeconomic backgrounds).

## 1. Introduction

Mental health problems affect 10% to 20% of children worldwide and are a leading cause of disability in young people [1,2]. For many, mental health problems begin in early childhood and persist into later childhood and adolescence [3,4], ultimately shaping a range of adult health and social outcomes [5,6,7]. As well as mental health difficulties, mental health also encompasses positive functioning or mental health ‘competence’ (e.g., pro-social behavior, respect for others, willingness to try new things) [8]. Promoting optimal mental health therefore requires a focus on reducing and preventing mental health difficulties while promoting mental health competence [9]. This more holistic approach to mental health promotion has become a core feature of recent global mental health promotion strategies and frameworks [7,10]. 

Environmental exposures early in life, such as the security of the child’s attachment to the primary caregiver, influence young children’s mastery of key skills (e.g., ability regulate one’s own emotions and find positive ways to deal with stress) that form the building blocks of optimal mental health later in life [11]. The processes that build these skills occur during specific windows of time (known as ‘sensitive periods’), such that mental health trajectories are set early. Early childhood mental health is linked to other important early childhood outcomes; for example, mental health in the early years has been associated with children’s early learning and school readiness [9,12]. Hence, early childhood has been identified as a key period for intervening to reduce later mental health problems and developmental inequities [7,11,13,14,15]. Intervening to promote mental health in the early years is more cost-effective than treating mental disorders later in life [16], allowing children to reach their full developmental potential [1]. 

For policymakers and public health practitioners, the need to reduce mental health inequities has been a primary focus. These differences in health outcomes arise not from biological differences between individuals, but from unequal access to resources and unequal exposure to health risks [17] and are seen in the early years, between and within countries. That is, consistently the most disadvantaged children have the worst mental health outcomes [15,18,19]. This signifies an opportunity to reduce child mental health inequities by changing the socio-spatial conditions in which children are raised (e.g., the resources available to children and the environments where they live). Both aspects of early childhood mental health (i.e., difficulties and competence) illustrate a social gradient—where each step in greater disadvantage is associated with a commensurate step up in difficulties and a step down in competence [20]. Importantly, disadvantage operating at both the family level (i.e., fewer family resources and lower maternal education) and neighborhood level (e.g., concentrated poverty) have been identified as significant determinants of inequities in early childhood [19,21]. These inequities are of major concern, as it becomes increasingly difficult to close these gaps as children age, with the gaps in developmental trajectories typically widening [11,15]. 

### 1.1. Conceptual Framework

Socio-ecological theory has long recognized that children develop in the context of multiple environments, including family and home environments, neighborhoods and schools, and broader society [22]. Individual- and family-level factors have been most widely studied, with known determinants of mental health inequities in early childhood including the child’s gender and age (individual-level), alongside maternal psychological distress, family income and socio-economic factors (e.g., employment), family structure, and parenting behaviors (family-level) [18,23,24]. Neighborhoods have long been recognized as key contexts in which children develop [25]. Several neighborhood qualities are known to be important to young children’s mental health and development, including socio-economic composition (e.g., concentration of poverty), social and institutional resources (e.g., capacity of local residents to intervene in neighborhood problems), and safety (e.g., neighborhood crime, incivilities like graffiti and vandalism) [25,26]. Socio-ecological theory understands these neighborhood features as interrelated and interacting with other individual- and family-level characteristics. For example, neighborhoods where residents are unlikely to intervene in local incivilities are likely to experience higher levels of disorder and crime, and this may have a disproportionate impact on the mobility of families who do not have private transportation and rely on walking or public transport. 

An important part of the neighborhood system is the built environment, which includes the physical features which are made or altered by humans, such as public parks, housing, and the local road network [27]. It should be noted that there is a relationship between the built environment and other socio-economic neighborhood characteristics; for example, there is evidence that more disadvantaged neighborhoods are more likely to have fewer parks and public open space than more advantaged neighborhoods [28]. However, the lack of parks and other built environment resources is not an inherent feature of disadvantaged neighborhoods, suggesting that this can be modified to promote health and prevent illness. Indeed, qualitative research from Australia found that in areas with similar socio-economic makeup (i.e., both relatively disadvantaged), aspects of the built environment (alongside other neighborhood factors) differentiated neighborhoods with better-than-expected child development outcomes from those with as-expected outcomes [29]. Within the ‘equigenic environments’ literature, the built environment has received attention as one possible platform to reduce inequities. The inequitable distribution of neighborhood built environment resources may be a key mechanism through which health inequities are perpetuated [28]. Hence, the provision of high-quality resources (e.g., safe, attractive spaces that cater to target groups) may be promising potential interventions to help reduce health inequities. 

**Figure 1:**
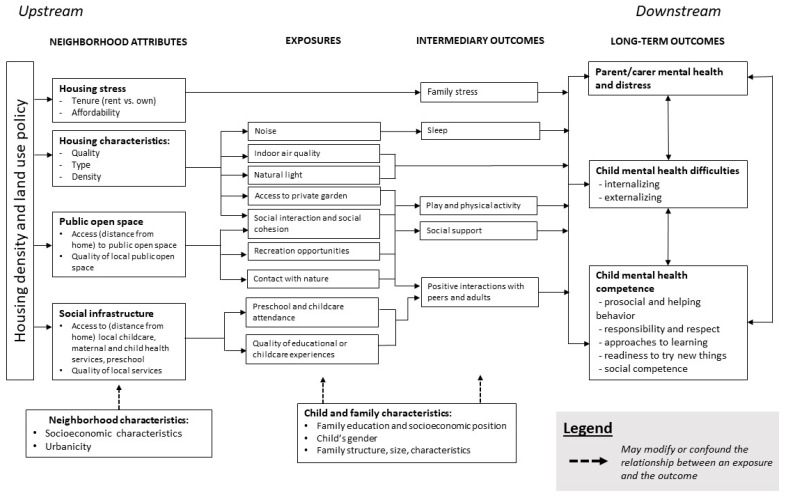
Conceptual framework theorizing possible pathways from the neighborhood built environment to child mental health outcomes.

There are a range of pathways through which the built environment may influence mental health in the early years (Figure 1). Based on findings from previous qualitative research [29], this review examines three domains of the neighborhood built environment that have been identified as important from an early childhood perspective: (1) nature and public open space; (2) local social infrastructure and service quality; and (3) housing (including residential density, housing typology and quality, and housing affordability). This review focuses on these domains in the context of urban environments. The plausible links between young children’s mental health and these three domains are briefly summarized below. 

### 1.2. Nature and Public Open Space

Several plausible mechanisms may explain the associations between access to nature and public open space (i.e., publicly accessible land, such as a park, that may be used for recreation purposes) and early childhood mental health. These include both direct effects on the child and indirect mechanisms (mediated by effects on their primary caregivers) [30]. Nature and public open space have been theorized to restore attention and reduce stress [31], and some evidence with older children suggests that living closer to public open space [32] and in neighborhoods with more nature [33] is associated with fewer mental health problems. A substantial body of evidence suggests that exposure to natural areas is a protective factor for adult mental health [34], and may indirectly shape young children’s mental health through promoting the mental health of their primary caregivers.

Access to high-quality public open space and nature (e.g., tree canopies, greenery and green spaces) in the neighborhood also provides children with important opportunities for physical activity and social interaction [35,36,37]. For example, qualitative research has found that young children consider public open space to be important to their social wellbeing, as it provides opportunities to play and be with their friends and other children [38]. The social role of these spaces may provide children with opportunities to build social competence, while also enabling social support systems for their caregivers. 

The availability of nature and high-quality public open space may not be equitably distributed amongst young children and families. There is some evidence that compared with advantaged areas, more disadvantaged neighborhoods have fewer [28] or lower-quality [39,40] public open spaces, which may discourage its use and restrict opportunities for promoting young children’s mental health. 

### 1.3. Local Service Access, Quality, and Social Infrastructure

The availability of key health, education, social and municipal services that are required for local living—termed ‘social infrastructure’—has received increasing attention as an important health-promoting neighborhood resource [41]. For young families, important services may include maternal and child health services, early childhood education and care, parent groups, playgroups, allied health services, and others. These services are crucial to young children’s wellbeing and development. For example, research has established that preschool attendance promotes positive mental health and reduces the likelihood of poor mental health outcomes, especially for socioeconomically disadvantaged children [23]. 

Yet, recent evidence shows that access to early childhood education services is not equal from one neighborhood to the next; children living in more disadvantaged neighborhoods typically have access to fewer and lower quality early childhood education services compared with their peers in more affluent neighborhoods [42]. Access to high-quality social infrastructure close to home may result in more families having the opportunity to use local services, therefore spending less time travelling and more time in their neighborhood, and having greater capacity to build neighborhood social cohesion [30].

### 1.4. Housing

Access to safe and secure housing has long been recognized as a social determinant of health and a fundamental human right [43,44]. Most of the literature around housing and young children’s mental health is focused on more proximal aspects of the home environment, such as the availability of stimulating learning materials in the home, disorganization or lack of home routines, and overcrowding. For example, housing ‘chaos’ has been conceptualized as a social determinant of young children’s health and wellbeing [45]. Chaotic home environments that are crowded and disorganized are theorized to increase exposure to noise, inhibit the establishment of family routines, and in-turn, increase stress on both caregivers and children [45]. While conceptualizations of housing chaos have tended to focus on family structure and parenting behaviors, scholars have suggested that the relationship between children’s mental health and their exposure to chaotic home environments may be closely intertwined with the built form of housing [46]. Indeed, more upstream aspects of neighborhood housing—such as its affordability, type, density (high-rise and residential density), and quality—may impact children’s mental health by setting the conditions for stable, secure, and stimulating home environments. For example, lack of affordable housing in the neighborhood may create uncertainty about housing security, increase stress on primary caregivers, and impact on parenting practices (thereby influencing young children’s mental health). 

Neighborhood housing may also impact on the behaviors of young families who live there; for example, neighborhoods with low residential density (i.e., those characterized by ‘urban sprawl’) that typically lack walking and cycling infrastructure may discourage residents from walking, which in turn, may reduce young families’ opportunities for chance social encounters. Families living in high-rise housing may venture outdoors less frequently; in neighborhoods with high concentration of high-rise housing, this may restrict opportunities to meet other families and build neighborhood support networks and social cohesion. 

Finally, the physical form or quality of housing, such as provision of natural light and outdoor space, may impact on young children’s mental health directly, as well as indirectly, through the mental health of their caregivers [47,48,49]. For example, access to a private garden may provide opportunities for play and positive interactions between children and their caregivers, but may also reduce the likelihood of using local parks and interacting with other children. As another example, families living in apartments may experience greater exposure to noise compared with families living in detached single-family homes; this may impact on young children’s mental health directly or indirectly by reducing the quality or duration of children’s and caregivers’ sleep. 

### 1.5. Research Gaps

‘Neighborhood effects’ research (i.e., studies examining the influence of neighborhood attributes on health and wellbeing) in relation to early childhood mental health is a relatively new area of study. Given the emerging evidence that the neighborhood built environment is an important feature of the conditions in which children grow, play, and develop, and a prominent feature of children’s accounts and conceptualizations of their own wellbeing [38], it is time to take stock of what is currently known and identify gaps to guide future research. In particular, there is a need to synthesize and evaluate the evidence for associations between young children’s mental health and the neighborhood built environment. To date, the authors are not aware of any reviews that have synthesized the evidence of associations between the neighborhood built environment and both aspects of mental health (i.e., mental health competence and difficulties) during early childhood. 

### 1.6. Project Aim

The aim of this study is to systematically search and critically review the quantitative literature investigating associations between the neighborhood built environment and young children’s mental health, with a focus on the three domains outlined above: nature and public open space, local social infrastructure and service quality, and housing. A better understanding of how these features relate to child mental health is critical—as the number of children and families living in cities increases, there is a need to ensure that all children have access to safe and supportive environments that promote optimal mental health outcomes for all children [50,51]. 

## 2. Materials and Methods

### 2.1. Search Strategy 

This review used a systematic search and review methodology, which is well-suited to relatively new research areas [52]. Systematic search and review methodology combines elements of systematic review methodology (i.e., the transparent, comprehensive database search) with critical review methodology (i.e., interpretive synthesis that aims to extend conceptual and/or methodological thinking) [52]. This article follows the Preferred Reporting Items for Systematic Reviews and Meta-Analysis (PRISMA) reporting checklist for systematic reviews [53]. This review was not prospectively registered.

The search was conducted in ProQuest Central and PubMed (including MEDLINE) databases from inception until 17 January 2019. ProQuest Central is a compilation of databases from various disciplines including (but not limited to) major databases in psychology and education (e.g., PsycINFO, PsycARTICLES, ERIC). The full search strategy including all search terms is reported in Appendix A. Articles were screened for inclusion by the first author first by title, then by abstract, and finally full text articles were reviewed. The reference lists of included articles were searched to identify additional articles. As a final check for comprehensiveness, combinations of keywords were searched in GoogleScholar to identify additional articles. 

### 2.2. Inclusion and Exclusion Criteria

Population: Children up to eight years of age. Studies were included if there were a mix of younger (<8 years) and older (>8 years) children and: (a) The results were stratified by age group, such that associations for young children could be isolated; or (b) analysis found no difference in results between younger and older children. General population-based samples were included; non-representative samples (e.g., in-patient clinical samples) were excluded.

Exposure: Any objective or subjective measure of: (a) neighborhood housing quality, density, typology, or affordability; (b) neighborhood public open space quality, quantity, or access (e.g., distance from residence to nearest public open space; proportion of land area that is public open space within a geographic area); (c) quality and/or accessibility (i.e., distance from residence) to local social, health, municipal, or early childhood education and care services. 

Comparison: No limits were placed on comparison groups (e.g., intervention and control groups).

Outcome: Any measure of at least one of the dimensions of competence (i.e., social competence; emotional maturity; prosocial behavior; self-regulation; responsibility and respect; approaches to learning; readiness to try new things) or at least one of the dimensions of difficulties (i.e., emotional symptoms; conduct problems; hyperactivity or inattention; peer relationship problems) or disorders (e.g., Attention Deficit and Hyperactivity Disorder). 

Setting: Urban populations. Studies were included if there were a mix of urban and rural populations and the results were stratified by urbanicity such that associations for urban populations could be isolated. Studies conducted in military bases, refugee camps, in-patient clinical settings, or exclusively rural settings were excluded. 

Other: English language, peer-reviewed quantitative studies, full text available online. 

### 2.3. Data Extraction 

Data were extracted by AA using a literature table. Data extracted included: author, year of publication, country, age of participants when outcomes were measured, sample size, study design used, exposure and outcome measures, main results, and statistical adjustments. 

### 2.4. Quality Assessment and Synthesis

Following systematic search and review methodology, findings were critically (narratively) synthesized. Critical synthesis is an interpretive process, where the results of the included studies are iteratively compared and analyzed with respect to study quality as well as conceptual contribution. Quality was assessed with respect to the key data extracted (adjustments used, study design, measurement of exposure and outcome). Due to the diversity of exposure and outcome measures used, the quality of the overall body of evidence for each exposure and outcome was not formally assessed or synthesized in a meta-analysis, as is common practice for full systematic reviews. 

## 3. Results

**Figure 2:**
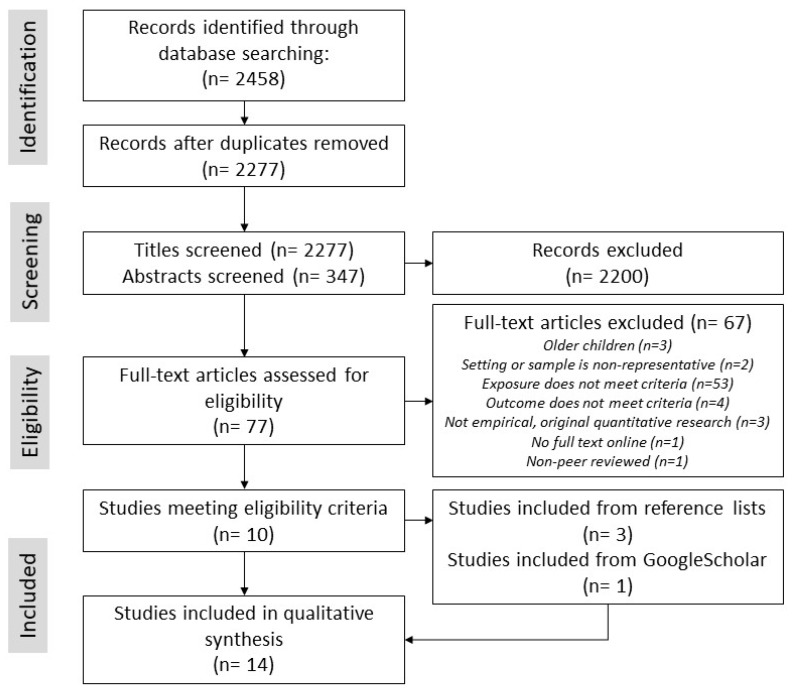
Preferred Reporting Items for Systematic Review and Meta-Analysis (PRISMA) flow diagram describing the procedure carried out to identify and assess articles for inclusion in the review.

After duplicates were removed the search strategy yielded 2277 published articles (Figure 2). After screening titles and abstracts, 2200 articles were excluded and 77 full text articles were assessed for inclusion. Of these, 10 met the inclusion criteria, and from the screening of reference lists of these articles, an additional three articles were assessed as meeting inclusion criteria. Finally, one article was identified from GoogleScholar as meeting inclusion criteria. In total, 14 articles were included in this review. 

### 3.1. Description of Included Studies

Fourteen studies examined associations between the neighborhood built environment and mental health in early childhood (Figure 2, Table 1). Most were cross-sectional (43%) or prospective cohort (43%) studies from high-income countries located in North America (USA), Oceania (Australia) and Europe (UK, Germany, Lithuania, Spain, Netherlands). Of these, nine studies examined aspects of housing quality, typology or density, while eight examined nature or public open space in the neighborhood. Only one study was identified that examined neighborhood social infrastructure and/or local service quality in association with early childhood mental health. Three studies examined neighborhood exposures in more than one built environment domain; all of these analyzed these exposures’ associations with mental health separately (rather than as a cumulative exposure). Nine studies defined mental health solely in negative terms (i.e., mental illness or difficulties), while two studies examined solely positive aspects of mental health. Three studies analyzed both negative and positive aspects of mental health; however, all three analyzed these as separate outcomes (i.e., using separate models for difficulties and pro-social behavior rather than a combined ‘optimal mental health’ profile). 

### 3.2. Nature and Public Open Space in the Neighborhood

Eight studies quantified associations between neighborhood nature and public open space and young children’s mental health difficulties (*n* = 5; 63%), competence (*n* = 1; 13%), or both (*n* = 2; 25%). Two studies (14%) used a case-control design, and the remaining studies were split evenly between cross-sectional or partial ecological designs (*n* = 6; 43%) and prospective cohort studies (*n* = 6; 43%). There were inconsistencies in the nature or public open space measures used. However, all studies examining measures of nature or public open space and their relationships with mental health difficulties reported at least one significant association in the theorized direction (i.e., higher exposure to nature or public open space associated with reduced difficulties). Two of three studies (67%) that examined measures of nature or public open space and mental health competence also reported at least one significant association in the theorized direction (i.e., higher exposure to nature or public open space associated with higher competence). For the articles examining nature or public open space, only two (29%) analyses (i.e., analyses of a second public open space measure within the same article) found associations in the opposite direction for mental health difficulties, and one study (33%) found associations in the opposite direction for mental health competence. 

Nature and public open space in the neighborhood were operationalized using diverse measures, ranging from measures of quantity (i.e., how much public open space is available in the neighborhood?) to access (i.e., how close does a child live to public open space?). In only one study, a measure of quality (e.g., how attractive is local public open space?) was also included. 

Availability (i.e., quantity) of nature or public open space in the neighborhood was measured in various ways including: Proportion of nature/public open space in a buffer area; satellite imagery around home; and parent-reported availability. Three studies used the proportion of natural space or public open space within a pre-defined area around a child’s residence [54,55,56]. In Australia, higher proportion of public open space in the child’s area of residence was associated with lower mental health difficulties [55]. In contrast, neither higher proportion of natural space or public parks were associated with difficulties in the United Kingdom, but higher proportion of natural space was associated with increased prosocial behavior [54]. Further, when stratifying by maternal education (low vs. high) and by gender, this study found significant differences between subgroups; for example, higher proportion of natural space was associated with fewer peer problems in young children with less-educated mothers, and more prosocial behavior in children with more-educated mothers [54]. In the United States, a higher proportion of tree canopy around children’s residence was associated with improved self-regulation and decreased behavior concerns, but not with peer or teacher relationships or children’s initiative to meet their own needs [56]. 

Two studies used satellite imagery or aerial photography to measure quantity of natural or public open space in the neighborhood [56,57]. The Normalized Difference Vegetation Index (NDVI) was used to operationalize the concept of ‘surrounding greenness’ (i.e., availability of nature or green space) within a certain buffer area around children’s homes [57]. Findings for NDVI differed between subgroups. One study found that higher NDVI (i.e., greener neighborhood) was associated with worse difficulties (higher total difficulties, higher conditional problems, higher hyperactivity) in young children with more-educated mothers, but for those with less-educated mothers, the association held only for conditional problems [57]. In the more-educated mothers group, higher NDVI was also associated with less prosocial behavior [57]. In a study from the United States, the proportion of impervious surface (e.g., concrete) around a child’s home was used as a proxy measure for lack of nature; this measure was not associated with young children’s competence (socio-emotional functioning) nor with difficulties (behavioral concerns) [56].

Finally, in Germany, children whose parents reported a lack of local parks had higher odds of borderline-to-abnormal difficulties, as well as higher odds of borderline-to-abnormal hyperactivity [58]. 

Studies measuring access to nature or public open space largely operationalized this concept as the distance from a child’s residence to the nearest public open space (usually, a park). A study from the United States found no association between young children’s mental health (testing both difficulties and competence) and the proportion of houses around children’s residences that had access to public open space within a half a mile or less [56]. However, findings tended to vary across subgroups within these studies. For example, in Lithuania living further from parks was associated with higher mental health difficulties in young children whose mothers had completed a lower level of education, but no association was found for the higher maternal education group [57]. In Wisconsin (United States), living further from parks was associated with higher odds of attention deficit and hyperactivity disorder (ADHD) diagnosis only in Eastern Wisconsin, and not in Milwaukee County, after adjusting for population density [59]. Contrary to the theorized direction of association, a partial ecological study from Australia found that a larger neighborhood median distance to the nearest park was associated with better social competence and emotional maturity; this also held for the associations between social competence and emotional maturity and larger distances to ‘attractive’ (i.e., higher-quality) parks [60]. 

Two studies examined associations between young children’s mental health and the quality of neighborhood natural or public open space. The Australian study above also reported that further neighborhood median distances to the nearest ‘attractive’ (i.e., higher-quality) park was associated with better social competence and emotional maturity [60]. In contrast, a second Australian study using parent perceptions of local parks as a measure of neighborhood park quality found that children whose parents reported poorer quality parks had higher difficulties, compared with those whose parents perceived their local parks were of higher quality [55]. 

### 3.3. Social Infrastructure and Service Quality in the Neighborhood

Only one study was identified examining associations between mental health in early childhood and local health, education, municipal or social service access (e.g., distance from residence) and/or quality (e.g. quality of nearest service, highest quality score within a given distance from residence). This study measured neighborhood median distances to the nearest services in a small area, including the following services: Child-center-based-care services, kindergartens, family support services, child health clinics, and playgroup venues [60]. Contrary to the anticipated direction of association, higher median distances to child-center-based-care services was associated with better social competence and better emotional maturity, while the rest of the associations were not significant [60]. 

### 3.4. Neighborhood Housing

Nine studies examined associations between mental health in early childhood and neighborhood housing quality (problems e.g., dampness, darkness; and amenities). Most studies examined measures of mental health difficulties as the outcome (n = 6, 67%), while two studies (22%) examined mental health competence only, and one study (11%) examined both competence and difficulties. Most of these examined aspects of housing quality (n = 7; 78%). Few studies examined housing typology (n = 2; 22%) and housing density (n = 2; 22%) in relation to young children’s mental health. One study examined multiple aspects (both typology and quality) of neighborhood housing. No studies examined associations between young children’s mental health and housing affordability. 

Housing quality measures were diverse, covering a range of quality aspects including dampness, lack of key infrastructure (toilets, heating, electricity, hot water), access to private gardens, and presence of gas appliances in the home. Housing quality was operationalized using field researchers’ observations of housing quality [63,65,66], census data [67], or parent-reported features of the home environment obtained from questionnaires [62]. Due to the various measures and aspects of housing quality used, most of the housing quality studies were not able to be directly compared; however, findings from two key groupings of housing quality (housing problems, housing amenities) are summarized below. 

Four studies measured housing problems (e.g., dampness, lack of heating or hot water, lack of electricity) with mixed findings. In Spain, persistent dampness in children’s room was associated with lower social competence, but no association was found with dampness at home in general, in the living room, or in parents’ room [62]. Housing problems (dampness, no hot water or electricity) were significantly associated with increased behavior problems and increased maternal depression in the United Kingdom, although this analysis was not adjusted for socioeconomic factors [65]. However, a second study from the United Kingdom found no association between mental health difficulties and housing problems at the neighborhood-level [67], and a study from the Netherlands found no significant association between young children’s difficulties and their physical home environments, operationalized using a composite 10-item measure [66]. 

Three studies measured housing amenities as an aspect of quality; amenities included the presence of gas appliances (observed by a field researcher) [63] and access to a private garden (parent self-report) [54,61]. Young children living in homes with two gas appliances had significantly higher odds of inattention difficulties and nearly-significantly higher odds of ADHD, compared with those with no gas appliances in the home [63]. In two studies from the United Kingdom, young children with access to a private garden had significantly lower difficulties compared with those without a private garden [54,61], although no change in difficulties over time was observed [54]. One of these studies found that the association varied according to maternal education, with lack of access to a private garden being associated with higher difficulties in children with less-educated mothers, but not those with more-educated mothers [54]. However, the authors also note that not having access to a private garden was associated with a greater worsening in difficulties over time for the higher maternal education group only [54]. This same study also found evidence of effect modification (i.e. differences between subgroups) by gender: Private garden access was associated with lower total difficulties in both boys and girls, but the types of difficulties associated with garden access differed (for boys, peer problems and conduct problems; for girls, hyperactivity) [54]. Focusing on positive aspects of mental health, an Australian study found that higher percentages of home yard space in the neighborhood were associated with better emotional maturity [60]. 

Only two studies investigated the relationship between young children’s mental health and housing typology. One study found that children living in high-rise buildings had significantly higher behavioral difficulties compared with the overall sample [61]. The other reported no significant differences in behavior problems when comparing children living in high-rise flats, low-rise flats, and detached houses [64]. 

Finally, two studies examined housing density in relation to young children’s mental health, using either objective density measures or suburban versus central city classification. An Australian study examined the association between neighborhood housing density (residential density) and early childhood mental health competence, reporting no significant association [60]. A study from the United States reported that children with a suburban address had higher odds of ADHD diagnosis compared with children with a central-city address, but only for the Milwaukee County subgroup [59]. 

No studies were identified that examined associations between young children’s mental health and housing affordability. 

## 4. Discussion

We systematically searched and reviewed the literature examining associations between mental health in young children and the neighborhood built environment, informed by a socio-ecological model of early childhood mental health. We reviewed the literature for three domains of the neighborhood built environment: nature and public open space, social infrastructure and service quality, and housing. These were previously identified as being important neighborhood-level factors for influencing early childhood development [29]. Findings showed an emerging body of evidence in the public open space domain and the housing domain. While the diversity of country contexts and measures used limits the comparability of study findings, there are some clear signals from the evidence base that the neighborhood built environment appears important for mental health in early childhood. This warrants further investigation. For example, nature and public open space in the neighborhood was consistently associated with lower mental health difficulties, and a smaller number of studies suggests its importance to mental health competence as well. Nevertheless, several significant gaps were identified which could advance current understandings of this new area of neighborhood effects research. These gaps include a lack of studies investigating relationships between early childhood mental health and: local social infrastructure and service quality; housing density, housing typology, housing affordability; and quality of neighborhood public open space. In addition, there was a lack of studies examining positive mental health outcomes. 

Beyond these gaps, some of the recent studies identified in this review have opened up new lines of inquiry with the potential to deepen understandings of how the neighborhood built environment could potentially modify mental health inequities in early childhood. As expected, the included studies reported a strong social gradient in young children’s mental health outcomes and in access to neighborhood built environment resources. While only three of the 14 studies compared associations across socio-economic strata, two studies found that public open space may be more important to the mental health of children from more socioeconomically disadvantaged backgrounds [57,61]. 

Similar to a recent systematic review of the green space—adult mental health relationship [34], this review found that most studies included in the nature and public open space domain reported a protective relationship with mental health difficulties in young children. This body of evidence is relatively new, with several of these studies [54,55,56,58] published in the past three years. 

A diverse body of studies examined aspects of housing in the neighborhood and young children’s mental health. The quality of the studies in the housing domain tended to be lower (e.g., analyses that did not control for key confounders like family socioeconomic status) and some studies dated back several decades. Study results were mixed, but this is also likely due to the diverse housing measures used. Like the public open space domain, most studies examined only mental health difficulties and it remains unknown whether the determinants and pathways for positive mental health are shared. 

This review should be viewed in the light of its limitations. First, only peer-reviewed articles published in English and available in full text were included; second, a full systematic review and meta-analysis was outside of the scope of this review, due to the heterogeneity of exposure and outcome measures used. Nevertheless, this review possesses important strengths, including the use of a systematic and replicable search strategy, a diverse and interdisciplinary compilation of databases, and a critical review and synthesis of the included literature that draws on a socioecological framework of early childhood mental health. 

### Future Research Agenda

There have been calls for greater attention to questions of ‘for whom’ neighborhoods matter most [68,69]. Some of the more recent studies have started to explore the potential of the built environment to modify inequities. These articles examined the differential associations between the neighborhood built environment and early childhood mental health across diverse subgroups of children (e.g. differences by gender, socioeconomic groupings). To advance these understandings, we argue that future studies should include in their aims an examination of how associations or experiences may differ (or converge) across diverse subgroups of children. As the neighborhood built environment has the potential to be modified at a population level, a promising direction for reducing early childhood mental health inequities is identifying the key neighborhood features that ‘level up’ (i.e., bestow the largest mental health benefit to the most disadvantaged children to narrow inequities [70]). At the same time, disparities in access to key built environment resources should be investigated to how these might contribute to the perpetuation or widening of mental health inequities. It is likely that the relationships between the built environment, neighborhood and family socio-economic characteristics, and young children’s mental health vary from one country or context to the next. Hence, future research should examine how key built environment features are being delivered across diverse cities and populations. This evidence is urgently needed to inform land-use planning, especially considering global trends towards increasing urbanization and children in cities [51]. 

A key direction for future research is investigating the importance of social infrastructure (e.g., maternal and child health services, early childhood education and care services) and local service quality in relation to young children’s mental health. This review identified only one study examining relationships between young children’s mental health and social infrastructure. This gap is significant, as attending such services is strongly linked to young children’s wellbeing and is especially important for children from disadvantaged families [22]. The included study’s findings were contrary to the expected direction of association (closer proximity to services was associated with worse mental health); however, this may reflect the importance of service quality in relation to children’s mental health outcomes. Recent evidence shows that children living in more disadvantaged neighborhoods are more likely to have access to fewer and lower quality early childhood education services close to home [39]. As young families tend to stay close to home and travel relatively short distances to such services [39,67], there is a need to understand whether the availability of high-quality services may be associated with better child mental health, and the potential impact of service availability and quality on child mental health inequities. 

While the findings here are based on a relatively small number of studies and should therefore be viewed as preliminary, the real-world implications are that place-based initiatives should consider how housing and land-use policies (e.g., the location of new affordable housing units; standards for housing and apartment quality) may be modified to support young children and families, especially those facing disadvantage. Evaluations of policies, programs, and where possible, natural experiments that seek to create supportive built environments for families will be useful in understanding how such interventions impact on mental health inequities. Such evaluations can also advance knowledge about how to modify the built environment while minimizing any unintended consequences of doing so (for example, increasing gentrification and consequently displacing families facing disadvantage) [71].

Similar to other reviews of built environment exposures [34,72], this review identified studies using a diverse range of built environment measures. As others have noted, the lack of consistency in built environment measures is a limitation of the evidence base that inhibits the comparability of findings [73]. Future studies should aim to use reproducible yet theoretically motivated measures of the built environment to examine relationships with young children’s mental health. With the greater availability of objective and spatial data, it is likely that there will be less reliance on self-reported measures of the built environment in the coming years. Qualitative studies may provide a deeper understanding of the ways that young children and families from diverse circumstances use and experience their neighborhoods. Qualitative studies are also needed to better understand the geographic scales that matter most to families with young children, and how these scales may vary according to the specific behaviors and built environment attributes examined. For example, qualitative mapping technologies, such as the digital mapping method known as softGIS, enable participants to map key services and destinations that they use [74,75]; these methods may help shed light on the geographic scales that are relevant to young children and families. These neighborhood scales and shapes may vary according to young families’ personal resources, such as car ownership. Finally, clinical studies (e.g., using biomarkers like cortisol to examine the role of stress) can help elucidate the mechanisms through which the neighborhood built environment may be associated with child mental health.

The studies examined in this review relied mainly on measures of mental health difficulties, rather than more holistic understandings of young children’s mental health. This omission of the positive dimensions of mental health limits our understanding of the determinants of optimal mental wellbeing during early childhood, which is a critical period of development [8,20]. This should be explored in future research and be considered in the design of studies; for example, measures of mental health competence are available through data linkage in Australia [9]. 

Current understandings of the ecology of young children’s mental health would benefit from a few key directions for future research, including addressing the research gaps above and examining how the built environment could be modified to reduce inequities in child mental health. Studies seeking to investigate the associations between the neighborhood built environment and child mental health should also look to the qualitative evidence for a given neighborhood attribute when developing a conceptual framework for analysis. 

## 5. Conclusions

To the authors’ knowledge, this is the first review that systematically and critically examined young children’s (0–8 years) mental health in relation to their neighborhood built environment. Results indicate that this is a small yet promising line of inquiry. This emerging body of evidence indicates that the neighborhood built environment may be important for reducing mental health difficulties and increasing mental health competence in young children. Some directions for future research were identified, alongside several gaps in the evidence. Notably, there is a need for more studies examining: (1) Associations with positive aspects of mental health (i.e., mental health competence); (2) the role of understudied neighborhood attributes like social infrastructure and service quality; and (3) the potentially differential associations between the neighborhood built environment and mental health in the early years, and the potential for modifications in the built environment to reduce mental health inequities. 

## Figures and Tables

**Table 1:** Quantitative studies included in this review.

Author (Year)	Country	*n*	Design	Exposure	Outcome	Age at Outcome	Adjustments	Results
More than one built environment domain (*n* = 3 studies; N/POS: *n* = 3, HOUS: *n* = 3, SERV: *n* = 1)								
Baumgardner et al. (2010) [59]	USA	50,463	Case-control	N/POS-1: Distance from child’s home address to closest park N/POS-2: Distance from child’s home to nearest waterway HOUS: Suburban home address (compared with central-city address)	MHD: ADHD diagnosis (cases) versus no diagnosis (controls)	5–17 years	Child’s age, child’s sex, child’s race, Eastern Wisconsin vs. Milwaukee County (stratified), population density, median household income (area-level)	N/POS-1 MHD: In Eastern Wisconsin, larger distance associated with higher odds of ADHD diagnosis (OR: 1.04, 95% CI 1.03–1.04). N/POS-2 MHD: In Eastern Wisconsin, NS. MHD: In Milwaukee County, larger distance to nearest waterway associated with lower odds of ADHD diagnosis (OR: 0.92, 95% CI 0.88–0.97). HOUS MHD: In Milwaukee County, suburban address associated with higher odds of ADHD (OR: 1.40, 95% CI 1.20–1.63).
Christian et al. (2017) [60]	Australia	149 NHs (23,395 children)	Partial ecological	N/POS-1: Population-based median distance to nearest park N/POS-2: Population-based median distance to nearest attractive park N/POS-3: Population-based median distance to nearest pocket park N/POS-4: Population-based median distance to nearest nature/conservation area SERV-1: Population-based median distance to nearest Kindergarten SERV-2: Population-based median distance to nearest child-center-based care SERV-3: Population-based median distance to nearest family support service SERV-4: Population-based median distance to nearest child health clinic SERV-5: Population-based median distance to nearest playgroup HOUS-1: % residential land not part of building footprint (home yard space in NH) HOUS-2: Residential density	MHC: Social competence (TR): Odds of poor social competence. MHC: Emotional maturity (TR): Odds of poor emotional maturity.	5 years	NH SES, % households in NH with 4 year old that were: female, Aboriginal or Torres Strait Islander origin, had one or more siblings, at least one parent > 24 years, single parent families, at least one parent educated beyond secondary school, family income < $3000/fortnight, moved in last 12 months	N/POS-1 MHC: Larger distance to nearest park associated with better social competence (OR: 0.996, 95% CI 0.993 to 0.999) and better emotional maturity (OR: 0.989, 95% CI 0.977 to 0.998). N/POS-2 MHC: Larger distance to nearest attractive park associated with better social competence (OR: 0.990, 95% CI 0.978 to 0.999). Emotional maturity: NS. N/POS-3 & N/POS-4 MHC: NS. SERV-2 MHC: Larger distance to child-center-based-care associated with better social competence and better emotional maturity. SERV-1, 3, 4, & 5 MHC: NS. HOUS-1 MHC: Higher % home yard space in NH associated with better emotional maturity (5th quintile OR: 0.745, 95% CI 0.567 to 0.969). HOUS-2 MHC: NS.
Richardson et al. (2017) [54]	Scotland	2909	Cross-sectional (by age 4 years); Prospective cohort (by age 6 years)	N/POS-1: % land area that is public parks around 500m (Euclidean) of child’s home postcode N/POS-2: % land area that is natural space around 500m (Euclidean) of child’s home postcode HOUS: Access to sole or shared private garden (yes/no) (PR)	MHD: Total difficulties (PR) MHC: Prosocial behavior Outcome at 6 years: change in MHD or MHC over time.	4 years, 6 years	Child’s sex, maternal education (stratified)	N/POS-1 MHD: Overall and in both maternal education groups: NS at 4 years, 6 years. MHC: Overall and in both education groups: NS at 4 years, 6 years. N/POS-2 MHD: Overall NS at 4 years, 6 years. MHD: In lower education group, higher % area natural space associated with fewer peer problems (−0.08 per IQR increase) at 4 years, NS at 6 years. MHC: Higher % natural space associated with more prosocial behavior at 4 years (+0.08 per IQR increase), NS at 6 years. MHC: In higher education group, % area natural space associated with more prosocial behavior (+0.12 per IQR increase). HOUS MHD: Overall no garden access associated with higher: total difficulties (+1.15), peer problems (+0.23), hyperactivity (+0.52), conduct problems (+0.27) at 4 years. MHD: Overall, change in difficulties over time (at 6 years): NS. In high education group, no garden access associated with worsening emotional problems and total difficulties over time (at 6 years), but NS for low education group. MHC: Overall: NS.
Nature and public open space domain (*n* = 6)								
Balseviciene et al. (2014) [57]	Lithuania	1,468	Cross-sectional	N/POS-1: Distance (straight line) from child’s home address to closest park N/POS-2: Residential greenness (average NDVI in 300m buffer around child’s home)	MHD: Total difficulties (PR) MHC: Prosocial behavior (PR)	4–6 years	Child’s sex, child’s age, maternal education (stratified), parenting stress	N/POS-1 MHD: In lower education group, larger distance associated with more total difficulties (β: 0.069, *p* < 0.05) and more peer problems, conditional problems, and hyperactivity. In higher education group, NS except for larger distance nearly associated with fewer emotional problems (β: −0.008, *p* < 0.1). MHC: In lower education group, larger distance associated with less prosocial behavior (β: −0.029, *p* < 0.05). Higher education group: NS. N/POS-2 MHD: In higher education group, higher NDVI associated with more total difficulties (β: 2.286, *p* < 0.1), and more conditional problems and hyperactivity. MHC: NS in low education group. In high education group, higher NDVI associated with less prosocial behavior (β: −1.104, *p* < 0.05).
Feng & Astell-Burt (2017) [55]	Australia	4968	Prospective Cohort	N/POS-1: % land area classified as parkland within child’s area of residence (SA-2). N/POS-2: Perception (PR) of good parks, playgrounds, play spaces in neighborhood (low quality vs high quality).	MHD: total difficulties, internalizing, externalizing (PR)	6–7 years (ages 4–5 years at baseline)	Child’s sex, child’s Indigenous status, child’s age group, NH SES, NH urbanicity	N/POS-1 MHD: Higher % parkland associated with lower difficulties (21 to 40% bracket: −0.29 (95% CI −0.47 to −0.10), and both lower internalizing and externalizing difficulties. N/POS-2 MHD: Poor quality parks associated with higher difficulties (disagree vs. agree: 0.53, 95% CI 0.34 to 0.72) and both higher internalizing and externalizing difficulties.
Flouri et al. (2014) [61]	England	6384	Prospective Cohort	N/POS: % land area that is green space	MHD: Emotional and behavioral problems (PR)	5 years, 7 years	Family SES (PR) (stratified), NH SES	MHD: Overall sample: NS. MHD: In low SES group, higher % green space associated with fewer emotional difficulties at 5 years.
Scott et al. (2018) [56]	USA	1551	Prospective Cohort	N/POS-1: % houses within 0.5 miles of public park (public, outdoor recreation area) around child’s home. N/POS-2: % impervious surface around child’s home. N/POS-3: % tree canopy around child’s home.	MHD: behavioral concerns (TR) MHC: Socio-emotional functioning (TR): Initiative, self-regulation, attachment	4–5 years	Child’s age, child’s race (Hispanic ethnic status), area-level median income, violent crime rate, population density, multilevel techniques (accounted for nested data).	N/POS-1 MHD: NS. MHC: NS. N/POS-2 MHD: NS. MHC: NS. N/POS-3 MHD: Higher % tree canopy associated with improved (less) behavior concerns (γ = −0.19, *p* < 0.01). MHC: Higher % tree canopy associated with improved self-regulation (γ = 0.175, *p* < 0.01).
Zach et al. (2016) [58]	Germany	5117	Cross-sectional	N/POS: Availability (PR) of public parks or green space in neighborhood (yes vs. no).	MHD: total difficulties and hyperactivity. Binary—classified as ‘normal’ vs. ‘borderline or abnormal.’ (PR)	Preschool, age not specified	Child’s sex, family SES, NH traffic load, crowding	MHD: In sample (unweighted) no access to green space was associated with higher odds of difficulties (OR: 1.92, 95% CI 1.72–2.96) and higher odds of hyperactivity-inattention (OR: 1.53, 95% CI 0.99–2.35). MHD: In weighted data (representative of Bavaria), no access to green space associated with higher odds of difficulties (OR: 3.17, 95% CI 1.76–5.70) and higher odds of hyperactivity-inattention (OR: 3.03, 95% CI 1.64–5.58)
Housing domain (*n* = 6)								
Casas et al. (2013) [62]	Spain	381	Prospective cohort	HOUS: Dampness in child’s bedroom, parent’s bedroom, living room, any other room in first 2 years of life (PR). Categorized as ‘never’, ‘ever (<2 years)’, ‘persistent (2 years)’.	MHC: Social competence (TR)	4 years	Child’s age, child’s sex, maternal education, maternal smoking during pregnancy, weeks of breastfeeding, folic acid intake during pregnancy, number of people living in the house, housing location (urban area, housing estate, country house)	MHC: Persistent dampness in child’s room associated with worse social competence scores (compared with non-persistent) (β: −6.54, 95%CI −12.19 to −0.89). MHC: Ever damp in child’s room (compared with never): NS. Dampness at home, in parent’s room, in living room: NS (irrespective of ever/never, persistent/non-persistent).
Morales et al. (2009) [63]	Spain	398	Prospective cohort	HOUS: Number (1 or 2) of household gas appliances (cooking, heating and cooling systems) compared with no gas appliances (HV)	MHD: ADHD (PR and TR). MHD: Inattention subset (PR and TR) MHD: Hyperactivity subset (PR and TR)	4 years	Child’s sex, maternal SES, maternal education, school trimester at testing, outcome evaluator (neuropsychologist), maternal smoking during pregnancy, number of smokers at home, maternal alcohol consumption during pregnancy, home location.	MHD: Two gas appliances associated with higher odds of inattention (OR: 3.59, 95% CI 1.14–11.33) and nearly associated with higher odds of ADHD (OR: 2.72, 95% CI 1.01–7.28) compared with no gas appliances. MHD: One gas appliance: NS.
Richman (1974) [64]	England	75	Cross-sectional	HOUS: Living in high-rise flats (higher than four floors), low-rise flats (not higher than four floors), houses (HV).	MHD: proportion in each group with behavior problems (PR and HV).	3 years	No statistical adjustments. Comparable SES between the three groups.	MHD: No significant differences between the three groups in proportion of behavior problems (high-rise flats: 16.0%, low-rise flats: 28.0%, houses: 20.0%).
Richman (1977) [65]	England	196	Case-control	HOUS-1: Living in high-rise flats. HOUS-2: Housing in poor condition (dampness, no electricity, no hot water) (HV).	MHD: behavior problems (PR and HV)	3 years	No statistical adjustments. Comparable SES between cases and controls.	HOUS-1 MHD: Children living in high-rise housing had significantly higher behavior problems (30% scored 10+, compared with 14% in overall sample, *p* < 0.01). HOUS-2 MHD: Of the children with a behavior problem and maternal depression, 27% (12/44) had severe housing problems, compared with 7% (5/75) with no behavior problem or maternal depression (*p* < 0.01).
Rijlaarsdam et al. (2013) [66]	Netherlands	2164	Prospective Cohort	HOUS: Physical home environment: 10-items including cleanliness, central heating system present, cluttered, dark, building is safe, play area outside is safe, neighborhood is pleasant.	MHD: internalizing and externalizing (PR).	3 years	Child’s sex, child’s age, child’s national origin (non-Western), family low income, maternal education, socio-emotional involvement with parent, maternal depressive symptoms at 20 weeks gestation.	MHD: NS.
Thompson et al. (1996) [67]	England	1047	Cross-sectional	HOUS: % households with ‘amenities’ (e.g., toilets, hot water) in the child’s ward of residence	MHD: behavior problem (yes/no), difficult temperament (yes/no), overactivity (yes/no) (PR)	3 years	No statistical adjustments.	MHD: NS.

Key: ADHD attention deficit and hyperactivity disorder. CI confidence interval. HOUS exposure/neighborhood attribute in the housing domain. HV obtained from trained observer during a home visit. IQR interquartile range. MHC mental health competence. MHD mental health difficulties. N/POS exposure/neighborhood attribute in the nature and public open space domain. NH neighborhood. NDVI Normalized Difference Vegetation Index. NS statistically non-significant findings. OR odds ratio. PR parent-reported. SA-2 Statistical Area-2 (on average, about 10,000 persons). SERV exposure/neighborhood attribute in the local social infrastructure and service quality domain. SES socioeconomic status. TR teacher-reported/assessed.

## Appendix A

### Pub Med (including MEDLINE) Search Strategy

((((“mental health”[tw] OR “complete state”[tw] OR (flourishing adj2 languishing)[tw] OR “wellbeing”[tw] OR “social development”[tw] OR “emotional development”[tw] OR “behavioural development”[tw] OR “behavioral development”[tw] OR “strengths and difficulties”[tw] OR “mental disorder*”[tw] OR “mental difficulties”[tw] OR “mental illness*”[tw] OR “conduct problem*”[tw] OR “emotional symptom*”[tw] OR “conduct disorder*”[tw] OR “hyperactiv*”[tw] OR “externali*”[tw] OR “internali*”[tw] OR “mental competenc*”[tw] OR “socioemotional”[tw] OR “socio-emotional”[tw] OR “prosocial*”[tw] OR “social competenc*”[tw] OR “self-regulation”[tw])) AND (“young child*”[tw] OR infant*[tw] OR toddler*[tw] OR “early years”[tw] OR “preschool*”[tw] OR “pre-school*”[tw] OR “kindergarten*”[tw] OR “0-8 year*”[tw] OR “younger age*”[tw] OR “young age*”[tw])) AND (“built environment*”[tw] OR “ecolog*”[tw] OR “neighbourhood*”[tw] OR “neighborhood*”[tw] OR “place-based”[tw] OR “green space*”[tw] OR “natural area*”[tw] OR “natural space*”[tw] OR “contact with nature”[tw] OR “public open space*”[tw] OR park[tw] OR parks[tw] OR “housing”[tw] OR “house*”[tw] OR “residen*”[tw] OR ((access OR proximity OR distance OR quality) adj2 (service* OR education OR “child care” OR childcare OR “general practice*” OR “health centre*” OR “health center*”)[tw])) AND (full text[sb] AND English[lang])

### ProQuest Central Search Strategy

(noft(“mental health” OR “complete state” OR (flourishing NEAR/2 languishing) OR “wellbeing” OR “social development” OR “emotional development” OR “behavioural development” OR “behavioral development” OR “strengths and difficulties” OR “mental disorder*” OR “mental difficulties” OR “mental illness*” OR “conduct problem*” OR “emotional symptom*” OR “conduct disorder*” OR “hyperactiv*” OR “externali*” OR “internali*” OR “mental competenc*” OR “socioemotional” OR “socio-emotional” OR “prosocial*” OR “social competenc*” OR “self-regulation”) AND noft(“young child*” OR infant* OR toddler* OR “early years” OR “preschool*” OR “pre-school*” OR “kindergarten*” OR “0-8 year*” OR “younger age*” OR “young age*”) AND noft(“built environment*” OR “ecolog*” OR “neighbourhood*” OR “neighborhood*” OR “place-based” OR “green space*” OR “natural area*” OR “natural space*” OR “contact with nature” OR “public open space*” OR park OR parks OR “housing” OR house* OR residen* OR ((access OR proximity OR distance OR quality) NEAR/2 (service* OR education OR “child care” OR childcare OR “general practice” OR “health centre*” OR “health center*”)))) AND la.exact(“English”) AND PEER(yes)
